# Surf’s Up for Postural Stability: A Descriptive Study of Physical Activity, Balance, Flexibility, and Self-Esteem in Healthy Adults

**DOI:** 10.3390/jfmk10030290

**Published:** 2025-07-29

**Authors:** Guillermo De Castro-Maqueda, Miguel Ángel Rosety-Rodríguez, Macarena Rivero-Vila, Jorge Del Rosario Fernández-Santos, Teppei Abiko

**Affiliations:** 1 Department of Physical Education, School of Education Science, University of Cádiz, Puerto Real, 11407 Cádiz, Spain; miguelangel.rosety@uca.es (M.Á.R.-R.); macarenariverovila@hotmail.com (M.R.-V.); jorgedelrosario.fernandez@uca.es (J.D.R.F.-S.); 2 Department of Physical Therapy, Faculty of Health Sciences, Kyoto Tachibana University, Kyoto 567-0031, Japan; abiko@tachibana-u.ac.jp

**Keywords:** aquatic sports, stability, flexibility, extensibility, self-esteem

## Abstract

**Background:** This study examines balance, flexibility and self-esteem among healthy individuals who engage in surfing compared to those who do not surf. **Methods:** A cross-sectional study design was conducted with 124 participants divided into the following groups: Group 1: Surfers *n* = 42; Group 2: individuals performing over 3 h of physical activity per week *n* = 43; and Group 3: individuals performing fewer than 3 h of physical activity per week *n* = 39. To assess balance, the Star Excursion Balance Test (SEBT) and the Flamenco Test (FBT) were used, the sit-and-reach test (SRT) was used to measure hamstring extensibility, the Rosenberg Scale was used to measure self-esteem, and the International Physical Activity Questionnaire (IPAQ) was used to measure physical activity levels. **Results:** Regarding descriptive characteristics, G1 participants were significant older than those of G2 and G3 (*p* < 0.05 and *p* < 0.001, respectively). Moreover, there was a higher proportion of females in G3 than in G1 and G2 (*p* < 0.05). The results revealed significant differences in balance between the surfers and those engaging in fewer than 3 h of activity per week (*p* < 0.05). G1 obtained significantly higher results in SEBT-left leg than G2 and G3 (*p* < 0.001) and higher result in SEBT-right leg and FBT than G3 (*p* < 0.05) but no significant differences in self-esteem were found. Significant differences in flexibility were observed between males and females (*p* < 0.001). **Conclusions:** This result suggests that surfing could have a positive effect on balance.

## 1. Introduction

Surfing is an aquatic sliding sport that demands muscular strength, balance, and flexibility, enabling the surfer to stay on the board and make the necessary adjustments according to the manoeuvres being performed and the behaviour of the wave [1].

As an outdoor sport, it not only benefits physical health but also mental health, promoting concentration, coordination, and stress reduction [2]. Exercising outdoors is associated with improvements in mental health [3], and the complex aquatic environment requires the development of postural control strategies involving visual, vestibular, and proprioceptive systems [4].

These capacities are essential for adapting the body to the anterior–posterior and lateral demands generated when manoeuvring on a surfboard in an unstable, slippery environment [1,4,5].

Postural control is an essential component of performing daily living activities. It relies on the central nervous system’s ability to integrate sensory inputs—including visual, vestibular, and somatosensory information—to generate a coordinated motor response [6].

This process involves complex biomechanical strategies and neuromuscular adaptations that stabilize body segments against gravity and maintain stability during upright stance. The vestibular system, in particular, provides information on the body’s orientation relative to the environment, influencing athletic performance and safety during both training and competition. It plays a key role in both static and dynamic balance [5,7].

Building on this, recent studies in healthy subjects have begun to assess how sport practice, in particular, repetitive balance training of competitive athletes for long periods, helps develop the plasticity of postural function by improving the proprioceptive, improving balance control [8].

Sports requiring constant balance also improve postural control, which is a limiting factor in performance, since no technical sports movement whether at an amateur or professional level can be efficiently executed without effective postural balance control [9].

Surfing additionally involves the dynamic nature of waves, requiring continuous adjustment, and demands prior experience and situational awareness for adequate balance strategies [1,4,5].

Moreover, the psychological benefits of physical activity suggest that sports such as surfing can positively influence self-esteem and overall wellbeing in children and adolescents [10,11,12]. In the context of rehabilitation, incorporating activities that concurrently provide both physical and psychological benefits may optimise treatment outcomes by fostering a more enjoyable exercise environment in natural settings, thereby reducing stress and improving self-perception [10,11,12,13].

Surfing may also play a pivotal role in preventing musculoskeletal injuries due to the physical demands of the sport, including significant strengthening of the core and stabilising muscles. These adaptations, combined with the inherent balance challenge in surfing encompassing both static and dynamic balance, may be beneficial for patients recovering from physical or psychological injuries [14,15].

The benefits of surfing are not limited to the physical realm; the marine environment in which it is practiced also contributes to improved mood and reduced anxiety levels, as confirmed by recent research into water sports [16,17,18]. Aquatic-based rehabilitation techniques have also been shown to improve both static and dynamic balance in adults [19].

This study aims to evaluate and compare the levels of balance among individuals who practise surfing and people with varying levels of physical activity. It also seeks to determine whether there is a direct relationship between sports practice and self-esteem, thereby assessing the potential role of surfing as a therapeutic tool in clinical contexts.

## 2. Materials and Methods

### 2.1. Participants

In this cross-sectional study, 138 healthy white individuals aged 18 to 55 were recruited. A total of 13 participants were excluded for not meeting the inclusion/exclusion criteria, resulting in a final total of 124 participants. Exclusion criteria for the total sample included the following: having any balance disorder, not fitting the established age range (2 participants), experiencing lower-limb musculoskeletal injuries in the past two years (3 participants), suffering from any major visual or auditory impairment (such as blindness, vertigo, or deafness), having an oncological or neurological illness, or taking medication that could affect balance (3 participants). Pregnancy was also an exclusion criterion, along with failing to complete the requested questionnaires fully (5 participants).

All participants were instructed not to perform any physical exercise within 24 h prior to the assessments to eliminate any potential influence on the results. They were informed of the study protocol and the associated risks/benefits, and they signed informed consent forms. They also retained the right to withdraw from the study at any time, although none chose to do so. All procedures were approved by an Institutional Ethics Review Committee, Ref. UCAGM 3/23 (University of Cádiz, Spain) in accordance with current national and international laws and regulations for human subjects (Declaration of Helsinki, Fortaleza, Brazil, 2013). All data were recorded anonymously and handled in strict compliance with Spanish data protection laws (Law 41/2002 of 14 November and Law 15/1999 of 13 December).

The participants were divided into three groups: Group 1 (G1): 42 surfers (31 men, 11 women; mean age = 29.7 ± 8.8 years; surfing experience = 11.05 ± 10.12 years). Inclusion/exclusion criteria for G1 required at least two years of surfing experience and at least 3 h of physical activity included surfing; the participants were permitted to practise other sports. Individuals with visual and/or auditory impairments (myopia or hypoacusis) were included in all the groups as long as those conditions were well managed.

Group 2 (G2): 43 individuals performing more than 3 h of weekly physical activity (28 men, 15 women; mean age = 25.0 ± 8.42 years). Inclusion criterion: practising a minimum of 3 h of physical activity per week, excluding surfing.

Group 3 (G3): 39 individuals performing fewer than 3 h of weekly physical activity (18 men, 21 women; mean age = 23.3 ± 5.6 years). Inclusion/exclusion criteria for G3 required that participants practise 3 or fewer hours of physical activity per week and not engage in surfing.

### 2.2. Measures

All measurements were taken in April 2024. Each participant attended one assessment session. They were contacted through emails sent to surf centres and schools, and via posters placed in universities, university schools, surf clubs, and various sports facilities. These emails provided detailed information about the tests and questionnaires. All instruments employed in this study have scientific validation.

Body Mass Index (BMI): Height and body mass were measured while the participants were barefoot, wearing shorts and a t-shirt. Height was measured to the nearest 0.1 cm using a stadiometer (Holtain Ltd., Crymych, Pembrokeshire, UK), and body mass was measured to the nearest 0.1 kg using a Seca scale (SECA 861, Leicester, UK). Instruments were calibrated for accuracy. BMI was calculated as body mass (kg) divided by stature squared (m^2^).

**Figure 1:**
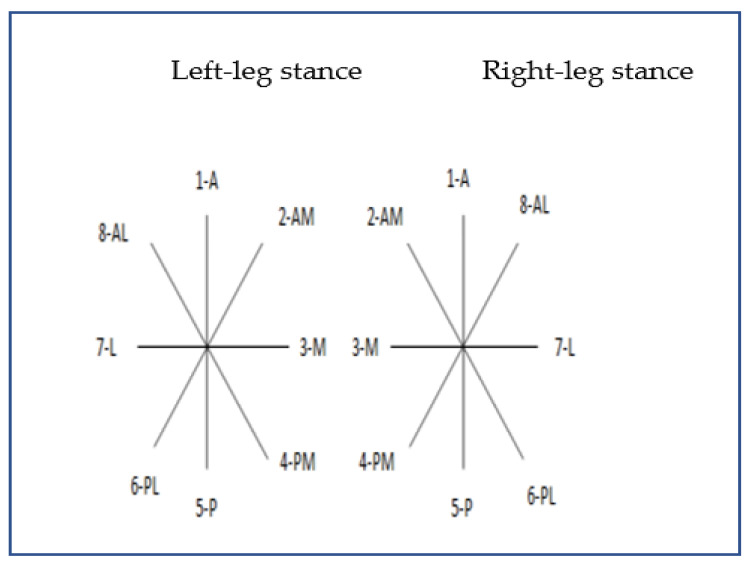
Note: 1-A. Anterior, 2-Am. Anteromedial, 3-M. Medial, 4-PM, Posteromedial, 5-P Posterior, 6-PL. Posterolateral, 7-L. Lateral, 8-AL. Anterolateral.

Star Excursion Balance Test (SEBT): This test is widely used to assess dynamic stability and proprioception, having been validated for various populations with high reliability [20,21,22,23]. Eight metric lines were placed on the floor, arranged like a star with 45° angles between them, each line ranging from 200 to 250 cm in length. Two calibrated lines were first placed in vertical and horizontal orientations (forming a symmetrical cross), followed by two diagonal lines in the shape of an “X”, all intersecting at the same central point. Each arm was labelled according to the direction in which the leg should move. Before starting the test, the length of both lower limbs was measured from the highest point of the anterior superior iliac spine to the centre of the fibular malleolus (Figure 1).

During the test, the participants stood at the intersection point of all lines on one leg (single-leg stance), keeping the supporting foot oriented towards the anterior direction of the star (1-A). The supporting foot remained in full contact with the floor without shifting or lifting the heel. With the other foot, the participants reached as far as possible, touching the floor lightly with the tip of the big toe in each of the eight directions, returning to the starting point before moving on to the next direction. When the tested foot was the left foot, the sequence began in the anterior direction and proceeded clockwise; with the right foot, it progressed anticlockwise. The farthest point reached was recorded, and this test was performed twice per leg, with a 2 min rest between trials.

To determine the final score, the mean of all measurement results was divided by 8 times the total leg length and then multiplied by 100 to obtain a percentage. Two quantitative variables thus emerged: the result for the left leg (SEBT left) and the result for the right leg (SEBT right)

$$SEBT=\frac{\mathrm{Mean}\;\mathrm{of}\;\mathrm{total}\;\mathrm{reach}\;\mathrm{distance}}{\left(\mathrm{Total}\;\mathrm{leg}\;\mathrm{length}\times 8\right)}\times 100$$

ref. [24] (Plisky et al., 2006).

Flamenco Test: This simple, reliable test assesses static balance and is used in both clinical and sports contexts [25]. The starting position involved placing one foot on the floor and the other on a 4 × 2 × 45 cm metallic board, secured at both ends by wooden supports, elevating it 8.5 cm above the floor. On a given signal, the participants adopted a single-leg stance on the board, bending the free leg and holding it with the hand on the same side. The stopwatch began running when the participant was balanced on the board and was stopped whenever they lost balance (with either foot contacting the ground). Each time balance was lost, the participant resumed the starting position, and timing continued until a total of 1 min on the board was reached. The number of falls from the board in that minute was recorded. If the participants fell more than 15 times in the first 30 s, the test was terminated. The test was performed twice with a 1 min rest between attempts.

Rosenberg Scale: Originally developed to assess self-esteem, this scale has shown utility in evaluating psychological factors and emotional wellbeing [26,27,28]. Each participant completed a validated questionnaire (10 items) prior to the balance tests. Of these 10 items, 5 are worded positively and 5 negatively, measuring the individual’s feeling of satisfaction with themselves.

For items 1 to 5, the responses from A to D were scored 4 to 1, respectively, while for items 6 to 10, the responses from A to D were scored 1 to 4. The results were interpreted as follows: 30–40 points indicates high self-esteem, 26–29 indicates average self-esteem, and below 25 indicates low self-esteem.

Physical activity and sports history: The level of physical activity was measured using the International Physical Activity Questionnaire (IPAQ, 2011) [29], which, among other items, queries the frequency and duration (at least 10 min per episode) of physical activity over the last seven days. The participants were also asked if they were members of any sports federation or had engaged in regular sports practice in the past two years. This questionnaire was supplemented with questions about surfing experience and weekly surfing frequency. Based on the information provided, each participant was classified as physically active or inactive.

Hamstring flexibility: This component was evaluated using the sit-and-reach test (SRT) following the established protocol [30]. This test has high validity and reliability [31,32] and is one of the most commonly used linear methods [33]. In the SRT, initially described by Wells & Dillon [34], the participants sat on the floor with their legs together and extended, and their feet flexed at 90° against a measurement box marked with a ruler, Fabrication Enterprises Inc., White Plains, NY, USA). The participants wore sports clothing (top and shorts) without shoes. With their palms facing downwards and fingers extended, they were instructed to reach forward as far as possible, sliding their hands along the ruler, holding the position for at least two seconds. The SRT score (in cm) was recorded as the final position reached by the fingertips on the ruler, with higher scores indicating better performance. The test was performed twice and the higher score was recorded [32,33,34].

### 2.3. Procedure

Upon arriving at the assessment area and prior to the balance and flexibility tests, the participants completed questionnaires on health habits, physical activity (IPAQ), and self-esteem (Rosenberg Scale). They also signed an informed consent form regarding their participation and permission to be recorded during the tests. All tests were conducted indoors under stable climatic conditions, with the ambient temperature between 22° and 24°. Each participant performed a short warm-up consisting of 5–10 min on a stationary bicycle or jogging.

Additionally, the participants completed a questionnaire on their age, sex, occupation, sports activities (type and duration), surfing experience, history of trauma or pathologies, unhealthy habits, medications taken, and any relevant personal history.

### 2.4. Data Analysis

Descriptive characteristics were reported as mean and standard deviation for quantitative variables and frequencies and percentage for qualitative variables. Data normality was assessed using the Shapiro–Wilk test for all quantitative variables. One-way ANOVA and chi-squared tests were conducted to examine differences in the descriptive characteristics between groups for quantitative and qualitative variables, respectively. ANCOVA was performed with age and sex as covariates to assess differences between groups in test results. RSES differences were modelled using a multinomial logistic regression with group category as the main predictor and age and sex as covariates.

All model assumptions were verified prior to analysis. When significant main effects were found, post hoc pairwise comparisons were performed using Bonferroni’s correction. A significance level of α = 0.05 was set for all hypothesis testing. All analyses were conducted using the R programming language for statistical computing (version 4.2.2).

## 3. Results

### 3.1. Participant Characteristics

A total of 124 healthy male and female aged 18 to 55 took part in this study (77 males). Participant characteristics in each category were classified as G1 (29.7 ± 8.8 years), with a mean of 11.05 ± 10.12 years of surfing experience; G2 (25.0 ± 8.42 years); and G3 (23.3 ± 5.6 years). The G1 participants were significantly older than those of G2 and G3 (*p* < 0.05 and *p* < 0.001, respectively). Moreover, there was a higher proportion of females in G3 than in G1 and G2 (*p* < 0.05).

Table 1 summarises the characteristics for the overall group. The mean (±SD) BMI values recorded were 24.0 ± 4.5 for the whole group, 24.1 ± 3.4 for female, and 23.8 ± 3.2 for male.

### 3.2. Balance Tests

For the test variables, G1 obtained significantly higher results in SEBT-left leg than G2 and G3 (*p* < 0.001) and higher results in SEBT-right leg and FBT than G3 (*p* < 0.05).

Regarding the Star Excursion Balance Test, the greatest reach distances and highest averages for each leg were recorded in the surfer group compared to the other two groups. In the Flamenco Test, higher scores correspond to more falls, which implies poorer performance. The G3 members exhibited the highest number of falls. Table 2 displays the descriptive results for each group and the different balance tests.

As regards the SEBT for the left leg, when comparing the three groups, no significant differences were found between G1 and G2, though differences did exist between G1 and G3. In comparing all three groups in the Flamenco Test, a significant difference emerged between surfers and both other groups (*p* < 0.05). The difference between G2 and G3 was not significant.

In addition, the relationship between the balance tests and age was examined to see if older or younger participants performed better or worse. The results showed no relationship between age and performance in both balance tests.

### 3.3. Hamstring Flexibility

G1 obtained the lowest performance in the flexibility test compared to the other groups, which may be attributed to the lower proportion of women in this group. When analysed by sex, women significantly outperformed men in the flexibility test (*p* < 0.001), despite men reporting higher levels of physical activity (*p* < 0.05). In the sit-and-reach test (SRT), women achieved a mean score of 5.3 ± 5.1 cm, while men scored 2.1 ± 5.3 cm. These differences may be explained by biological or hormonal factors associated with sex. No significant positive correlations were found with other measured variables.

### 3.4. Self-Esteem

With regard to the Rosenberg Self-Esteem Scale, 88.0% of participants in Group 1 exhibited high self-esteem, 10.5% demonstrated average self-esteem, and none presented with low self-esteem. In G2, 84.2% had high self-esteem, 15.7% had average self-esteem, and none had low self-esteem. In G3, 84.21% had high self-esteem, 10.5% had average self-esteem, and 5.2% had low self-esteem. No differences were found across groups, nor was any association detected between sports practice and self-esteem.

## 4. Discussion

The present study links the practice of surfing to a significant improvement in both dynamic and static balance, compared to other populations and different sports disciplines. The findings indicate that sports requiring continuous adaptation to unstable environments strengthen postural control, as is the case in surfing, snowboarding, and ice hockey [1,4,35,36,37]. These observations are supported by studies describing the physiological demands of surfing, emphasising not only the aerobic and anaerobic capacity required but also the importance of effective postural control to maintain balance on the board in constantly changing aquatic conditions [38].

Constant exposure to unstable surfaces promotes proprioception and intermuscular coordination, encouraging more advanced postural strategies that could be harnessed in therapeutic contexts. The results demonstrate a significant difference in the SEBT (dynamic balance) between people who surf and those engaging in fewer than three hours of physical activity weekly. Notably, within the group performing under three hours of exercise per week, 12 participants did not perform any physical activity. Meanwhile, all participants in the surfer group also practised another sport, supporting the idea that better balance in this group is due to both surfing and regular physical activity. This aligns with a published study indicating that physical activity improves both static and dynamic balance compared to inactivity [39].

No significant differences in dynamic balance were found between the group performing more than three hours of physical activity per week and the group performing fewer than three hours. This suggests that if practising any sport were the sole contributor to better balance, one would expect significant differences between these two groups. Hence, it is likely that the practice of surfing leads to improved dynamic balance.

Research has shown that proprioception, strength, and power are key components in aquatic sports like surfing, where ongoing exposure to unstable surfaces enhances intermuscular coordination and reflex responses [1,4,5,40]. Surfing also exerts a positive impact on specific populations, with its physical, psychological, and social benefits demonstrated by extensive intervention programmes [8,9,41]. Additionally, aquatic physiotherapy research has documented similar improvements albeit in a less challenging setting for rehabilitation, achieving significant advances in postural control and general mobility of the treated area [42,43,44].

With respect to balance, studies have compared dynamic balance, measured by the Star Excursion Balance Test, among hockey and football players. For instance, Bhat and Ali-Moiz [45] found no significant differences between the two groups, suggesting that better balance may be linked to sports participation in general rather than the specific type of sport. This was also observed in our study, where the surfing group reported engaging in another sport as well.

However, focusing on the development of dynamic lower-limb strength, the SEBT appears to be a solid adjunct for detecting bilateral performance differences, possibly indicating the efficacy of surfing training in reducing lower-limb asymmetry and improving postural control [46].

Furthermore, Reed et al. suggested that regular outdoor sports practice may help alleviate stress [12,47], a benefit that also appears in surfing through its combination of physical exercise and contact with nature. This highlights the potential of surfing not just as a recreational pursuit but also as a therapeutic tool. It not only aids balance and coordination but also provides a relaxing environment that may counteract chronic stress.

Regarding self-esteem, measured by the Rosenberg Scale, most participants scored in the high range; so, this study did not find a relationship between sports practice and self-esteem. These findings are consistent with the work of Molina-García et al. [48], who likewise found no significant differences between physically active and inactive participants for both sexes. Compared to a study by Monteiro et al., no significant differences in self-esteem were observed between those who did and did not practise dance [49]. Nevertheless, surfing could contribute to reducing depressive symptoms, which, in turn, may be related to improvements in self-esteem [50].

As for hamstring extensibility, our study found that women surfers exhibited greater flexibility than their male counterparts. These data match other studies using the SRT [31,32,33], and this difference could be due to biological or hormonal factors related to sex. However, we did not observe significant differences with other variables.

Our findings indicate that longitudinal studies are warranted to assess the long-term benefits of surfing across different populations. Future studies might explore how to combine surfing with other conventional aquatic therapies to maximise proven benefits [43,44]. Investigations should also delve into the psychological mechanisms that enhance balance and self-esteem and consider designing personalised interventions for specific clinical groups.

## 5. Conclusions

In conclusion, this study confirms that practising surfing and physical activity improves both dynamic and static balance in healthy individuals. However, within the age ranges studied, no definitive relationship was found between age and balance performance. Regarding self-esteem, no differences were detected between surfers and other participants, though further research is needed to corroborate this finding. Moreover, the potential therapeutic applications of surfing for populations with balance disorders were highlighted.

Our results suggest that this approach could offer a solid foundation for future research and for integrating surfing into innovative rehabilitation protocols. Surfing may serve as a complementary technique in rehabilitative treatments for patients with balance disorders, and further studies should explore these benefits in clinical settings.

## 6. Limitations

This study has several limitations worth noting. Firstly, its observational, cross-sectional design limits the ability to establish causal relationships between surfing practice and improved balance. Longitudinal or experimental designs would provide a clearer picture of how surfing influences balance and whether this effect persists over time. Secondly, the participants were recruited from a single geographical region, which may affect the generalisability of the findings to broader or more diverse populations. Thirdly, self-reported data on physical activity (e.g., hours of exercise per week) could introduce recall bias or inaccuracies that affect the reliability of group classification.

Furthermore, the environmental variability in surfing conditions (for example, wave height, water temperature, or currents), gender differences and the balance of participants were not standardized, which could influence the magnitude of balance adaptations. Finally, although the age range was fairly broad (18–55), the sample did not include individuals with clinically diagnosed balance disorders or other relevant comorbidities, limiting the application of these results to more specialised or clinical populations. Future research addressing these limitations, such as by conducting randomised controlled trials, recruiting more diverse cohorts, and systematically monitoring environmental factors, would help confirm and extend the conclusions drawn here.

## 7. Practical Applications

Despite these limitations, the findings offer several practical applications. Firstly, surfing-based exercise programmes may be considered as an adjunct to traditional balance training for healthy adults, given the positive effect observed on dynamic and static balance. Health and fitness professionals could incorporate surf-specific drills or balance-related manoeuvres (e.g., simulation training on unstable surfaces) into broader exercise regimens to enhance postural control.

Secondly, sports coaches and athletic trainers can use surf-like training environments (e.g., surf simulators, balance boards) to diversify their athletes’ conditioning routine, potentially improving proprioception and postural stability.

Thirdly, the data suggest that long-term participation in an activity requiring dynamic postural adjustments (such as surfing) might confer benefits in daily tasks that involve balance and coordination, which could be particularly relevant for injury prevention in other sports.

Finally, although not tested directly in clinical populations, the results hint that surf-related activities may hold therapeutic potential for individuals undergoing rehabilitation for mild balance disorders. Engaging in enjoyable physical activities could facilitate greater adherence to rehabilitation protocols and potentially accelerate improvements in postural control.

## Figures and Tables

**Table 1:** Descriptive characteristics of the sample.

Variables		All (n = 124)	G1 (n = 42)	G2 (n = 43)	G3 (n = 39)
Age (years)		26.1 ± 8.2	29.7 ± 8.8 ^a^	25.0 ± 8.4 ^b^*	23.3 ± 5.6 ^b^***
Sex (n (%))	Male	77 (62)	31 ^a^	28 ^a^	18 ^b^*
	Female	47 (38)	11	15	21
Height (cm)		170.3 ± 11.7	174.3 ± 11.7	169.5 ± 10.8	170.2 ± 11.8
Weight (kg)		69.0 ± 8.8	69.8 ± 9.0	66.5 ± 9.8	71.1 ± 6.8
BMI (kg/m^2^)		24.0 ± 4.5	23.2 ± 4.3	27.6 ± 4.7	24.9 ± 4.4

Different superscript letters indicate significant differences between groups. BMI = Body Mass Index. * *p* < 0.05; *** *p* < 0.001.

**Table 2:** Test results by experimental group.

Test		G1 (n = 42)	G2 (n = 43)	G3 (n = 39)
Sit-and-Reach Test (cm)		2.7 ± 6.2	3.7 ± 6.4	4.6 ± 5.6
SEBT—left leg		15.1 ± 2.2 ^a^	13.4 ± 1.5 ^b^***	12.6 ± 2.3 ^b^***
SEBT—right leg		14.2 ± 1.9 ^a^	13.3 ± 1.7 ^ab^	13.0 ± 1.8 ^b^*
Flamenco Balance Test (falls)		3.4 ± 3.1 ^a^	8.4 ± 5.5 ^ab^	12.4 ± 5.8 ^b^*
RSES (n (%))	High	37 (88)	36 (84)	33 (85)
	Moderate	5 (12)	7 (16)	4 (10)
	Low	0 (0)	0 (0)	2 (5)

SEBT indicates Star Excursion Balance Test; RSES, Rosenberg Self-Esteem Scale. Different superscript letters indicate significant differences between groups. * *p* < 0.05; *** *p* < 0.001.

## Data Availability

The raw data supporting the conclusions of this article will be made available by the authors on request.
